# Supplementary material for: PGPB Isolated from Drought-Tolerant Plants Help Wheat Plants to Overcome Osmotic Stress
Source: Plants (Basel). 2024 Nov 30;13(23):3381. doi: 10.3390/plants13233381 (PMC11644311; doi:10.3390/plants13233381)
Supplement: Supplementary file 1 [file plants-13-03381-s001.zip › plants-3315542-supplementary.pdf]

Table S1. Molecular identification of PGPB.

Isolate	Identity	Nearest Homolog Sequences (Accession Number)	Accession Number
Cap03D	99.71%	*Arthrobacter agilis*	KF318389.1
	99.56%	*Arthrobacter pityocampae*	KR476452.1
	99.56%	*Arthrobacter ruber*	KR476452.1
Cap07D	99.93%	*Bacillus safensis*	MT642941.1
	99.93%	*Bacillus pumilus*	MT279500.1
	99.93%	*Bacillus velezensis*	MT492019.1
	99.93%	*Bacillus australimaris*	MT510169.1
	99.93%	*Bacillus wiedmannii*	MT239515.1
	99.93%	*Bacillus aerophilus*	OP986381.1
Cap09D	99.93%	*Bacillus safensis*	MT256302.1
	99.93%	*Bacillus pumilus*	MT367713.1
	99.93%	*Bacillus aerophilus*	OP986029.1
	99.93%	*Bacillus australimaris*	MH169000.1
App11D	100 %	*Bacillus pumilus*	CP054310.1
	100 %	*Bacillus zhangzhouensis*	MN826587.1
	100 %	*Bacillus safensis*	CP090354.1
Cap286	100 %	*Paenibacillus hordei*	MN629116.1
	100 %	*Paenibacillus nuruki*	MH707258.1
	100 %	*Paenibacillus chinensis*	PP725389.1
	100%	*Paenibacillus nicotianae*	OR121761.1
	100%	*Paenibacillus kyungheensis*	CP117416.1

Notes: Strains (*Arthrobacter* sp. Cap 03D, *Bacillus* sp. Cap 07D, *Bacillus* sp. Cap. 09D and *Paenibacillus* sp. Cap 286) were isolated from white pigweed (Chenopodium album L) and *Bacillus* sp. App 11D was isolated from seeds of camel thorn Alhagi pseudoalhagi (Bieb.) Fisch.

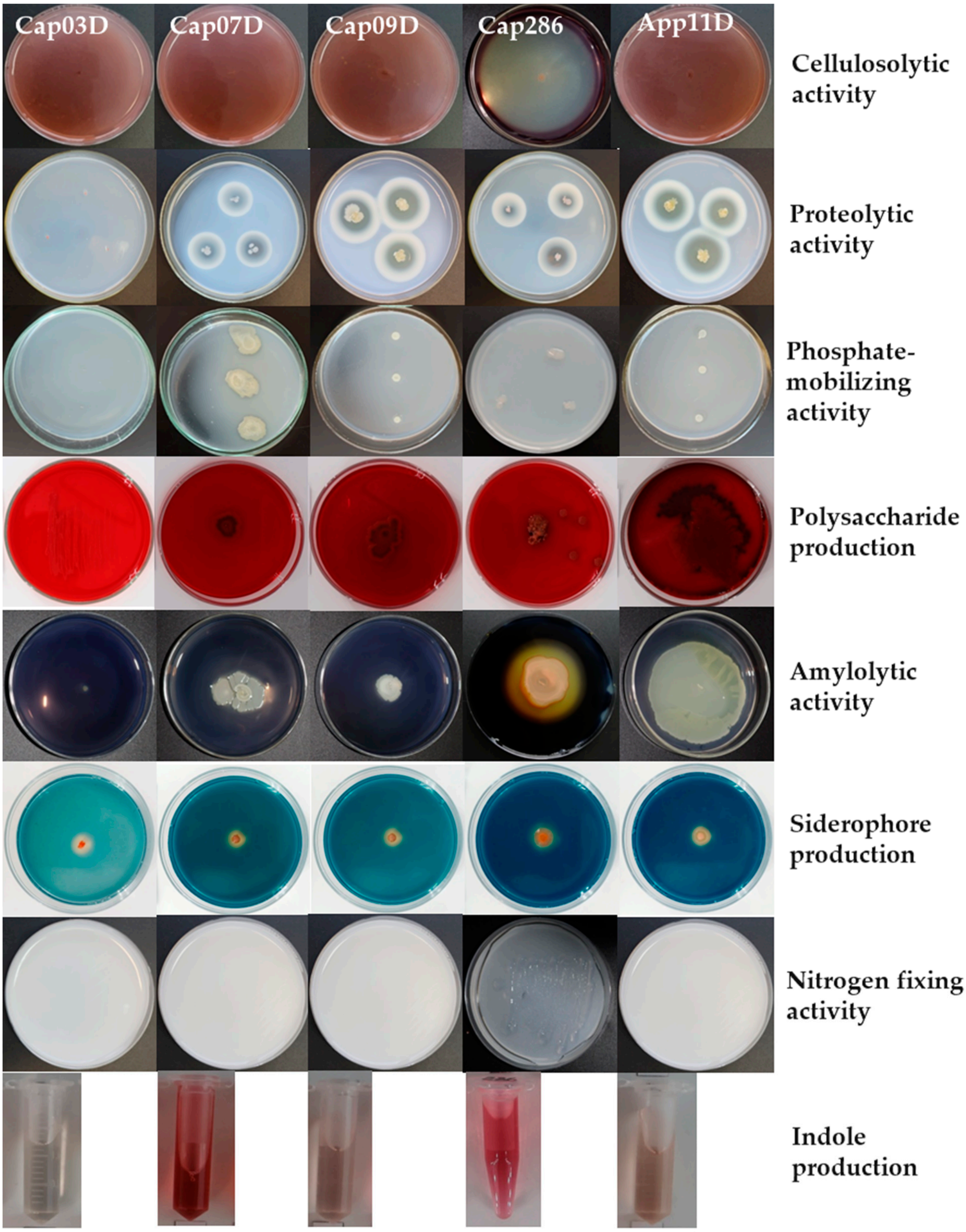

Figure S1. Metabolic activities of PGPB isolated from drought tolerant plants

Notes: The metabolic activities of each indicated strain are located in the column below. Strains (*Arthrobacter* sp. Cap 03D, *Bacillus* sp. Cap 07D, *Bacillus* sp. Cap. 09D and *Paenibacillus* sp. Cap 286) were isolated from Chenopodium album L and *Bacillus* sp. App 11D was isolated from seeds of *Alhagi pseudoalhagi. L.*

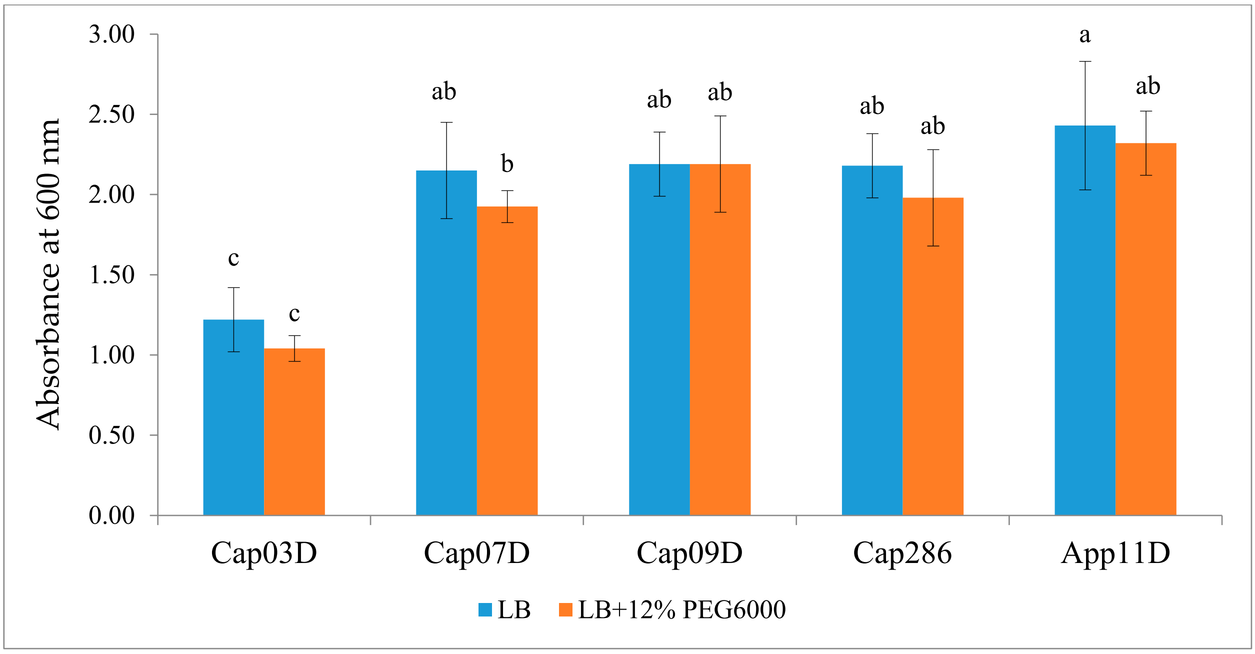

Figure S2. Effect of 12% PEG6000 on the bacterial growth.

Notes: LB –growth of corresponding strain on LB medium,LB+12% PEG growth of corresponding strain on LB medium Bars show ±SEM. Values in columns followed by different letters are significantly different at $p \leq 0.05$, as determined by Duncan's multiple range test.:

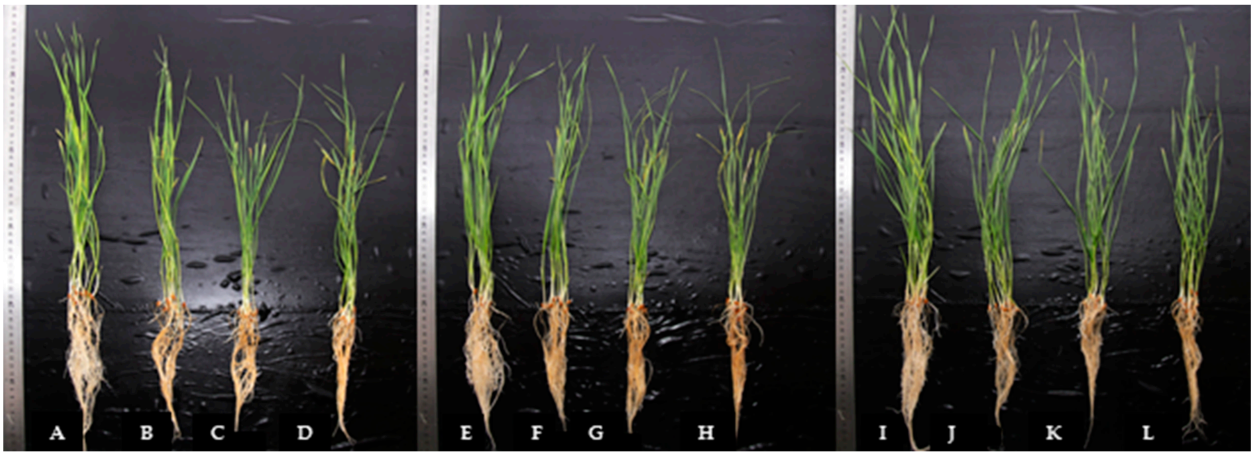

Figure S3. Effect of different concentrations of PEG 6000 (14 days of vegetation and 7 days of osmotic stress sexposure) and the inoculation with PGPB on growth of wheat plants cv. Leningradskaya 6.

Notes: A: control wheat plants growing on Knop solution; B: wheat plants growing on Knop solution with addition of 10%PEG 6000; C: wheat plants growing on Knop solution with addition of 12% PEG6000; D: wheat plants growing on Knop solution with addition of 16% PEG; E: wheat plants, growing on Knop solution and inoculated with *Arthrobacter* sp. Cap 03D;F: wheat plants growing on Knop solution with addition of 10% PEG 6000 and inoculated with *Arthrobacter* sp. Cap 03D; G: wheat plants growing on Knop solution with addition of 12% PEG 6000 and inoculated with *Arthrobacter* sp. Cap 03D; H wheat plants growing on Knop solution with addition of 16% PEG 6000 and inoculated with *Arthrobacter* sp. Cap 03D; I: wheat plants, growing on Knop solution and inoculated with *Bacillus* sp. App 11D; J: wheat plants growing on Knop solution with addition of 10% PEG 6000 and inoculated with Bacillus sp. App 11D; K: wheat plants growing on Knop solution with addition of 12% PEG 6000 and inoculated with Bacillus sp. App 11D; L: wheat plants growing on Knop solution with addition of 16% PEG 6000 and inoculated with with Bacillus sp. App 11D
